# Low-Level Expression of MTUS1 Is Associated with Poor Survival in Patients with Lung Adenocarcinoma

**DOI:** 10.3390/diagnostics11071250

**Published:** 2021-07-13

**Authors:** Seungyun Jee, Hyunsung Kim, Seongsik Bang, Yeseul Kim, Ha Young Park, Seung Sam Paik, Jongmin Sim, Kiseok Jang

**Affiliations:** 1 Department of Pathology, Hanyang University College of Medicine, Seoul 04763, Korea; jee.seung.yun@gmail.com (S.J.); hhnt5841@gmail.com (H.K.); grypony@naver.com (S.B.); sspaik@hanyang.ac.kr (S.S.P.); 2 Department of Pathology, Asan Medical Center, Seoul 05505, Korea; coabee@hanmail.net; 3 Department of Pathology, Busan Paik Hospital, Inje University College of Medicine, Busan 47392, Korea; pmint00@naver.com; 4 Department of Pathology, Anam Hospital, Korea University College of Medicine, Seoul 02841, Korea

**Keywords:** lung cancer, adenocarcinoma, MTUS1, immunohistochemistry, prognosis, public data

## Abstract

Microtubule-associated tumor suppressor 1 (MTUS1) is thought to be downregulated in arious human cancers, which suggests its role as a tumor suppressor. This study investigated the clinicopathological significance of MTUS1 expression in lung adenocarcinoma. Tissue microarray blocks consisting of 161 cases were constructed, and immunohistochemical staining was used to assess MTUS1 expression. Correlations of MTUS1 expression and clinicopathological parameters were analyzed. In addition, we used public databases and performed bioinformatics analysis. Low level of MTUS1 was significantly associated with higher clinical stage (*p* = 0.006), higher tumor stage (*p* = 0.044), lymph node metastasis (*p* = 0.01), worse histologic grade (*p* = 0.007), lymphovascular invasion (*p* = 0.014), and higher Ki-67 proliferation index (*p* < 0.001). Patients with low MTUS1 expression also showed shorter disease-free survival (*p* = 0.002) and cancer-specific survival (*p* = 0.006). Analysis of data from the Cancer Genome Atlas confirmed that the mRNA expression of MTUS1 in lung adenocarcinoma was significantly lower than that of normal lung tissue (*p* = 0.02), and patients with decreased MTUS1 expression showed significantly shorter overall survival (*p* = 0.008). These results suggest that MTUS1 may be a potential biomarker for predicting clinical outcomes in lung adenocarcinoma patients.

## 1. Introduction

Lung cancer is one of the most common causes of cancer death in both men and women [1]. In particular, the incidence rate of adenocarcinoma, the most common histologic subtype, has been increasing internationally [2]. Lung cancer is typically diagnosed at an advanced stage, and the prognosis of lung cancer remains poor despite recent advances in early detection and novel therapeutic agents. The 5-year survival rate for lung cancer is low at 18% for all stages combined and 5% for patients diagnosed at a distant stage [1]. The tumorigenesis of lung cancer is thought to be the result of complex genetic and environmental interactions. The roles of various genes as oncogenes or tumor suppressors are under investigation.

Microtubule-associated tumor suppressor 1 (MTUS1) is a potential tumor suppressor protein encoded by the *MTUS1* gene (also known as mitochondrial tumor suppressor gene 1, *MTSG1*), first reported by Seibold et al. in 2003 [3]. *MTUS1*-encoded proteins are classified as ATIP1, ATIP3 (ATIP2, ATIP3a, and ATIP3b), and ATIP4, each with different tissue specificity (ATIP; angiotensin II (AT2) receptor-interacting protein) [4]. ATIP1 and ATIP3 are thought to be the major splice variants, and are associated with cancer formation [5]. ATIP1 physically interacts with AT2 receptor, but whether ATIP3 interacts with AT2 receptor is unknown. Both ATIP1 and ATIP3 are known to interfere with the activation of extracellular signal-regulated kinase (ERK) signaling, thereby inducing cancer cell apoptosis [6,7,8]. ATIP3 also impairs microtubule dynamics causing prolonged mitosis and downregulates Snai2 and Vimentin while upregulating E-cadherin, thus interfering with epithelial to mesenchymal transition [9,10].

Downregulation of MTUS1 has been demonstrated in various human cancers, including pancreatic [3], ovarian [11], head and neck [7,12], colorectal [13], breast [14], bladder [15], stomach [16], lung [17], gallbladder [18], and kidney (renal cell carcinoma) [19] cancers. The precise role and clinical significance of MTUS1 in lung adenocarcinoma are unclear. In this study, we investigated MTUS1 expression by immunohistochemical staining and its association with clinicopathological factors in 161 lung adenocarcinoma patients. In addition, survival analyses were performed to assess the prognostic significance of MTUS1. Finally, public data from the Cancer Genome Atlas (TCGA) were analyzed.

## 2. Materials and Methods

### 2.1. Patients and Tumor Samples

In total, 184 consecutive cases of patients who underwent curative surgery for primary lung adenocarcinoma between January 2003 and December 2014 at Hanyang University Hospital in Seoul, Korea were studied retrospectively. All patients had undergone surgery, including wedge resection, segmentectomy, lobectomy, bilobectomy, or pneumonectomy, with or without lymph node dissection. None of the patients had received preoperative therapy. Of the 184 cases, 23 (12.5%) were excluded due to inadequate FFPE samples, and statistical analyses were performed in 161 cases.

Clinicopathological data were obtained from medical records and histopathological reports, and an additional review of the archived pathologic slides was performed. The clinicopathological parameters included patient age, sex, tumor size, T stage, N stage, 8th American Joint Committee on Cancer (AJCC) stage [20], histologic grade, pleural invasion, lymphovascular invasion, and perineural invasion. Histologic grading was based on both architectural patterns and nuclear features. In terms of the 2015 WHO classification, grade 1 cases corresponded to lepidic-predominant (well-differentiated), grade 2 cases to acinar- or papillary-predominant (moderately differentiated), and grade 3 cases to solid- or micropapillary-predominant (poorly differentiated) subtypes. Disease-free survival (DFS) was calculated from the date of operation to the date of recurrence or the last follow-up visit. Cancer-specific survival (CSS) was calculated from the date of operation until the time of death (excluding patients who died from causes unrelated to lung adenocarcinoma), or the last follow-up visit.

### 2.2. Tissue Microarray (TMA) Construction

Hematoxylin and eosin-stained slides were reviewed under light microscopy, and non-necrotic representative portions of the carcinoma were carefully selected. Single 2.0 mm sized tumor cores were punched out from each paraffin block and assembled into a recipient paraffin block with a manual TMA instrument (Unitma, Seoul, Korea). Then, 4 μm thick sections were obtained from the TMA blocks.

### 2.3. Immunohistochemical Staining

Immunohistochemical staining of the sections was carried out with a fully automated slide preparation Benchmark XT System (Ventana Medical Systems Inc., Tucson, AZ, USA). Primary antibodies against MTUS1 (1:100; polyclonal rabbit, Aviva, San Diego, CA, USA) and Ki-67 (1:100, ab16667 monoclonal rabbit, Abcam, Cambridge, MA, USA) were used according to the manufacturer’s instructions.

### 2.4. Interpretation of Immunohistochemical Staining

**Figure 1:**
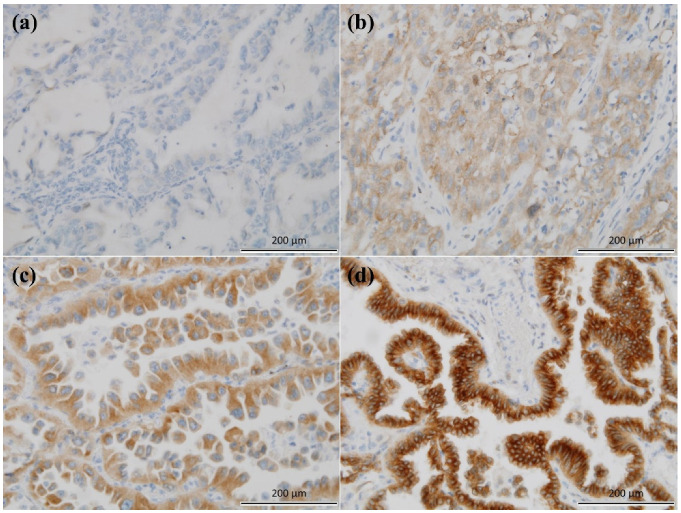
Microtubule-associated tumor suppressor 1 (MTUS1) immunostaining in lung adenocarcinoma (×200). (**a**) Negative, (**b**) weak, (**c**) moderate, and (**d**) strong.

Antibody expression was assessed using the H-score, as has been reported previously [21,22,23]. Cells showing strong, intermediate, weak, and no membranous or cytoplasmic staining for MTUS1 were scored as 3+, 2+, 1+, and 0, respectively (Figure 1). The proportion of tumor cells at each staining intensity was determined by eyeball estimation. The H-score was calculated as follows: H-score = [1 × (% cells 1+) + 2 × (% cells 2+) + 3 × (% cells 3+)]. Patients were divided into two groups according to the level of MTUS1 expression, low (H-score ≤ 130) and high (H-score > 130), by the receiver operating characteristics (ROC) curve maximizing Youden index, using disease-free survival. The proportion of tumor cells showing nuclear staining for Ki-67 proliferation index at any intensity was determined by eyeball estimation. The samples were all assessed while blinded to the clinicopathological findings and clinical outcomes.

### 2.5. Statistical Analysis

For statistical analysis, SPSS software (version 21.0; SPSS, Chicago, IL, USA) was used. The chi-square test was used to evaluate the associations of MTUS1 expression and various clinicopathological parameters. The Mann–Whitney U test was used for analysis of continuous variables with non-normal distribution. The Kruskal–Wallis test was used to compare the mean MTUS1 expression of different groups, with pairwise Wilcoxon test adjusted by the Benjamini–Hochberg (BH) method. The Kaplan–Meier survival curves for DFS and CSS were plotted, with the log-rank test performed to establish the level of significance. The Cox proportional hazard regression model was used to evaluate the prognostic significance of individual parameters. A *p*-value of <0.05 was accepted as indicating statistically significant results.

### 2.6. TCGA Data Analysis

In total, 479 lung adenocarcinoma cases were found in the TCGA dataset. We downloaded the clinical information and transcriptome profiles from the Genomic Data Commons (GDC) data portal [24]. We compared the mRNA levels of MTUS1 between lung adenocarcinoma and normal tissues. To identify the best separation value for dividing the samples into two groups, we performed a log-rank test for expression level in fragments per kilobase million (FPKM). The FPKM with the lowest log-rank *p*-value was selected, and samples were divided into low- and high-expression groups. The Kaplan–Meier survival curve for overall survival was plotted.

## 3. Results

### 3.1. Patient Characteristics

The clinicopathological features of the 161 lung adenocarcinoma patients are summarized in Table 1. The age of the patients ranged from 34 to 81 years, with a mean of 62.2 years. In total, 74 male and 87 female patients were included. Most patients received lobectomy (*n* = 130, 80.7%). Furthermore, 82 patients (50.9%) received surgery only, while 79 patients (49.1%) received additional therapies. The tumor size ranged from 0.2 cm to 13.0 cm, with a mean of 2.9 cm. Most cases were designated as histologic grade 2 (*n* = 110, 68.3%), 31 patients (19.3%) as grade 1, and 20 patients (12.4%) as grade 3. Ki-67 proliferation index ranged from 5 to 80%, with a mean of 15.7%. Pleural invasion was identified in 50 cases (31.1%), lymphovascular invasion in 65 cases (40.4%), and perineural invasion in 28 cases (17.4%). T stage distribution was as follows: 4 cases (2.5%) were assigned Tis, 74 (46.0%) T1, 68 (42.2%) T2, 8 (5.0%) T3, and 7 (4.3%) T4. Lymph node metastasis was found in 48 cases (29.8%). Following the 8th AJCC staging system, stage I was the most common (*n* = 94, 58.4%), while 4 patients (2.5%) were assigned stage 0, 32 (19.9%) stage II, and 31 (19.3%) stage III. The median follow-up periods for DFS and CSS were 24.58 and 30.02 months, respectively. Overall, 45 patients (28.0%) showed relapse, and 44 patients (27.3%) died due to lung adenocarcinoma.

### 3.2. Correlations and Comparison of Means between MTUS1 Expression and Clinicopathological Parameters in Lung Adenocarcinoma

A total of 161 patients were divided into two groups based on the level of MTUS1 expression: MTUS1—high (H-score > 130; *n* = 87) and MTUS1—low (H-score ≤ 130; *n* = 74). The results for the correlations between MTUS1 expression and clinicopathological parameters are summarized in Table 2. Low MTUS1 expression was significantly associated with larger tumor size (*p* < 0.001), worse histologic grade (*p* = 0.007), presence of lymphovascular invasion (*p* = 0.014), higher T stage (*p* = 0.044), higher N stage (*p* = 0.010), and more advanced 8th AJCC stage (*p* = 0.006). No significant association was shown between MTUS1 expression and age, sex, pleural invasion, or perineural invasion. In addition, low MTUS1 expression was significantly associated with higher Ki-67 proliferation index (*p* < 0.001).

The mean H-score among the three histologic grades was significantly different (*p* < 0.001), with significant differences between each pair of groups (*p* = 0.044 between grades 1 and 2; *p* < 0.001 between grades 2 and 3; *p* < 0.001 between grades 1 and 3), and the boxplot is shown in Supplementary Figure S1. The mean H-score among AJCC stage groups (0, 1A1, 1A2, 1A3, 1B, 2, and 3) was significantly different (*p* = 0.026) but pairwise comparisons showed no significance between any two groups. The median of MTUS1 expression was the highest in stage 1A1 group (median = 260.0, interquartile range 180.0–295.0, *n* = 11), followed by stage 0 group (median = 220.0, interquartile range 190.0–255.0, *n* = 4). The boxplot is shown in Supplementary Figure S2.

### 3.3. Prognostic Value of MTUS1 Expression in Lung Adenocarcinoma

**Figure 2:**
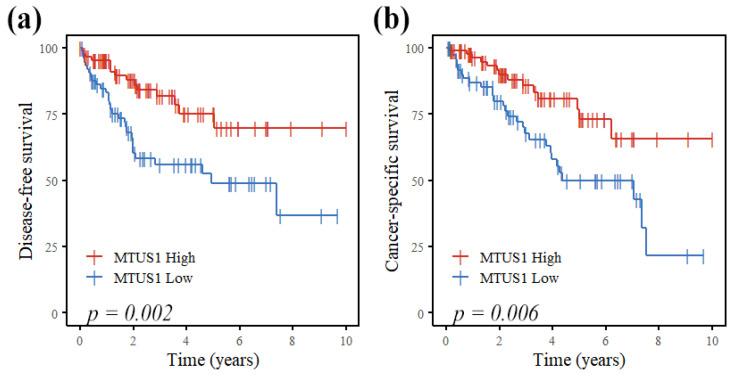
Cumulative disease-free (**a**) and cancer-specific (**b**) survival curves according to MTUS1 expression by immunohistochemistry in lung adenocarcinoma patients. There was significant difference in both disease-free and cancer-specific survival (Kaplan–Meier method with log-rank test).

As shown in Figure 2, patients with decreased MTUS1 expression showed less favorable prognoses, for both DFS (*p* = 0.002) and CSS (*p* = 0.006). Univariable Cox regression analysis revealed MTUS1 expression (high vs. low) as a significant prognostic factor (*p* = 0.003 and *p* = 0.007, respectively) for DFS and CSS. Other significant predictors included histologic grade (1, 2, and 3), pleural invasion, lymphovascular invasion, perineural invasion, T stage (Tis, T1 vs. T2, T3, T4), lymph node metastasis, AJCC stage (0, I vs. II, III), and Ki-67 proliferation index (Table 3). MTUS1 expression was not an independent prognostic factor in multivariable Cox regression analysis, whereas tumor size and lymphovascular invasion were independent prognostic factors for both DFS and CSS, and AJCC stage for CSS only.

Within the lung adenocarcinoma patient cohort, low MTUS1 expression was significantly associated with poorer prognoses for both DFS and CSS, in early-stage group (AJCC stages 0 and I, *n* = 98; *p* = 0.002 and *p* = 0.007 for DFS and CSS, respectively) and in surgical treatment-only group (*n* = 82; *p* = 0.011 and *p* = 0.006 for DFS and CSS, respectively). The difference in prognosis in terms of DFS and CSS according to MTUS1 expression was not significant in the late-stage group (AJCC stages II and III, *n* = 63; *p* = 0.92 and *p* = 0.86 for DFS and CSS, respectively) and in the adjuvant therapy group (*n* = 79; *p* = 0.12 and *p* = 0.37, respectively). The survival curves according to MTUS1 expression in early and late AJCC groups and in surgery-only and adjuvant therapy groups are shown in Supplementary Figures S3 and S4, respectively.

When the patients were divided into three groups according to MTUS1 expression (H-score 0–100: low group, *n* = 61; 101–200: medium group, *n* = 47; 201–300: high group, *n* = 53), the survival analysis revealed the low group with poorer prognosis, but the difference between the high and medium group was unclear, with overlapping curves (figure not shown).

### 3.4. TCGA Data Analysis

**Figure 3:**
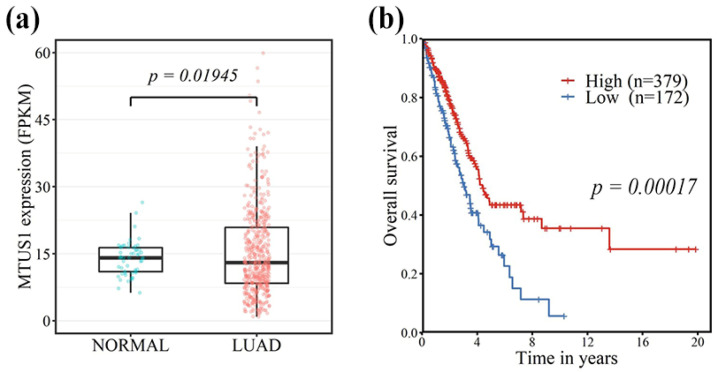
Data analysis from the Cancer Genome Atlas (TCGA) showing significantly lower mRNA expression of MTUS1 in lung adenocarcinoma than in normal lung tissue (**a**). In addition, there was a significant difference in overall survival according to MTUS1 mRNA expression (Kaplan–Meier method with log-rank test) (**b**).

Analysis of data from the TCGA confirmed that the mRNA expression of MTUS1 in lung adenocarcinoma was significantly lower than that of normal lung tissue (*p* = 0.02) (Figure 3a). Moreover, patients with decreased MTUS1 expression showed significantly shorter overall survival (*p* = 0.00017) (Figure 3b).

## 4. Discussion

*MTUS1* was discovered as a potential tumor suppressor gene, and downregulation of MTUS1 has been confirmed in several types of human cancers [3,11,13,14,15,16,17,18,19,25]. In this study, we showed that MTUS1 expression was significantly lower in lung adenocarcinoma tissues with adverse clinicopathological features, and we confirmed that the prognosis in terms of DFS and CSS was significantly worse in patients with lower MTUS1 expression, especially in patients at early stages and without additional treatment. MTUS1 was independently a significant prognostic factor according to univariable Cox regression analysis. In the multivariable analysis, MTUS1 was no longer significant, and rather acted as a confounding variable, as MTUS1 expression itself was already significantly associated with the other strong predictors.

To our knowledge, this study is the first to evaluate MTUS1 expression in tissue samples from a sizable cohort of lung adenocarcinoma patients. Our findings are consistent with results from studies of other human cancers and suggest that MTUS1 as a tumor suppressor may also play a crucial role in lung adenocarcinoma, with the potential to serve as a novel biomarker for lung adenocarcinoma patients, especially in early stages. Our findings are also consistent with results from the TCGA dataset, which showed lower MTUS1 mRNA expression in lung adenocarcinoma tissues compared with that of normal lung tissues and demonstrated shorter overall survival in lung adenocarcinoma patients with lower MTUS1 expression.

Significant correlations between low MTUS1 expression and pathologic T stage and histologic grade (differentiation) have also been unanimously reported in oral tongue squamous cell carcinoma [12], salivary adenoid cystic carcinoma [7], bladder cancer [15], gallbladder carcinoma [18], and renal cell carcinoma [19]. Other reported significantly associated clinicopathological parameters in these studies include tumor size [15], clinical stage [7,12,18], lymph node metastasis [12], and lymphovascular invasion [18]. Poor survival in patients with low MTUS1 expression was also reported in these studies [7,12,15,18]. In our study of lung adenocarcinoma patients, low MTUS1 expression was significantly associated with larger tumor size, higher T stage, lymph node metastasis, higher AJCC stage, worse histologic grade, lymphovascular invasion, and higher Ki-67 proliferation index. Of note, a higher Ki-67 proliferation index is known to be correlated with worse clinical outcomes in non-small cell lung cancer patients, including lung adenocarcinoma patients [26,27]. Furthermore, patients with decreased MTUS1 expression showed worse disease-free and cancer-specific survival.

Specifically, low MTUS1 expression was associated with clinicopathological parameters related to cancer proliferation (tumor size and Ki-67 proliferation index) and metastasis (lymphovascular invasion and lymph node metastasis) in our study. These findings are in line with the currently speculated role of *MTUS1*-encoded proteins (ATIPs) in tumor progression. The ATIP1 and ATIP3 splice variant of *MTUS1*-encoded proteins have been reported to interfere with ERK signaling, inducing apoptosis in cancer cells [6,7,8,9,10]. ATIP3 co-localizes with microtubules and may lead to prolonged mitosis [9,10], while also downregulating Snai2 and Vimentin and upregulating E-cadherin, leading to reduced migration and metastasis [7,8].

Interestingly, the mean H-score for MTUS1 expression among three histologic grades was progressively lower with poorer grades in our study. However, the level of MTUS1 expression at different points of cancer progression has not yet been specified. In fact, in the TCGA lung adenocarcinoma data, some cancer tissue exhibited higher MTUS1 expression than in normal tissues. Furthermore, in a study by Louis et al., prostate cancer was associated with increase in the *MTUS1*/ATIP mRNA expression (specifically ATIP1 and ATIP3 isoforms), compared to normal tissues and cell lines [28]. The authors suggest that re-expression or upregulation of ATIP may occur at an early phase in the malignant process, as a type of response to injury. In our study, the median of MTUS1 expression was the highest in stage 1A1 group, even higher than that of stage 0 group. Due to limited sample size, the data must be interpreted with caution, and validation with a larger cohort and additional normal tissue samples are warranted in the future.

Several studies have further investigated the function of MTUS1 using various lung cancer cell lines. Previous cell proliferation and migration studies with the A549 lung cancer cell line have confirmed the potential role of MTUS1 as a tumor suppressor [17,29]. In a study by Wescott et al., which investigated the mutational landscapes of KRAS-driven lung cancer, knockdown of *MTUS1* expedited growth in mouse lung cancer cell line driven by KRAS G12D [30]. The possible mechanisms of MTUS1 regulation are also being investigated. Parbin et al. showed that inhibiting DNA methylation with 5-aza-2′-deoxycytidine in the A549 cell line resulted in enhanced MTUS1 expression, suggesting that MTUS1 may be regulated by DNA methylation [29]. In addition, Gu et al. have shown that microRNAs miR-19a and miR-19b cooperatively repress MTUS1 expression to promote lung cancer cell proliferation and migration [17]. The role of microRNAs in controlling MTUS1 expression has also been studied in breast [14] and colorectal cancers [13]. Such regulatory mechanisms of MTUS1 expression represent an interesting area of study.

Our study was retrospective in nature, with a limited number of patients from a single institution, and we investigated MTUS1 expression by immunohistochemistry in human tissue samples only. Despite these limitations, our data demonstrated strong associations of MTUS1 expression level with established poor prognostic factors and survival in lung adenocarcinoma patients. The findings were further supported by results from the TCGA dataset analysis. In the future, specific downstream targets of *MTUS-1*-encoded proteins and regulatory mechanisms of MTUS1 expression may be further investigated.

In conclusion, decreased MTUS1 expression in lung adenocarcinoma patients was significantly correlated with adverse clinicopathological factors and poor disease-free and cancer-specific survival, especially for patients at early stage and with surgical treatment only, suggesting that MTUS1 may be a potential biomarker for predicting clinical outcomes in lung adenocarcinoma patients.

## Figures and Tables

**Table 1:** Clinicopathological characteristics of the studied lung adenocarcinoma patients.

Clinicopathological Characteristics		Number of Patients (*n* = 161, %)	
Age (years old)	34–81; mean 62.2	161	(100)
Sex	Male	74	(46.0)
	Female	87	(54.0)
Operative procedure	Lobectomy	130	(80.7)
	Segmentectomy	9	(5.6)
	Wedge resection	13	(8.1)
	Bilobectomy	3	(1.9)
	Pneumonectomy	6	(3.7)
Treatment	Surgery only	82	(50.9)
	Adjuvant therapy (chemotherapy and/or radiation therapy)	79	(49.1)
Tumor size (cm)	0.2–13.0; mean 2.9	161	(100)
Histological grade	Well (Grade 1)	31	(19.3)
	Moderate (Grade 2)	110	(68.3)
	Poor (Grade 3)	20	(12.4)
Ki-67 proliferation index (%)	5–80; mean 15.7	154	(95.6)
Pleural invasion	PL0	111	(68.9)
	PL1	39	(24.2)
	PL2	10	(6.2)
	PL3	1	(0.6)
Lymphovascular invasion	Present	65	(40.4)
	Absent	96	(59.6)
Perineural invasion	Present	28	(17.4)
	Absent	133	(82.6)
T stage	Tis	4	(2.5)
	T1	74	(46.0)
	T2	68	(42.2)
	T3	8	(5.0)
	T4	7	(4.3)
*n* stage	N0	113	(70.2)
	N1	25	(15.5)
	N2	21	(13.0)
	N3	2	(1.2)
8th AJCC * stage	0	4	(2.5)
	I	94	(58.4)
	II	32	(19.9)
	III	31	(19.3)
Recurrence	Recurrence	45	(28.0)
	No recurrence	116	(72.0)
Cancer-specific death	Death	44	(27.3)
	Alive	117	(72.7)

* AJCC: American Joint Committee on Cancer.

**Table 2:** Correlation between microtubule-associated tumor suppressor 1 (MTUS1) expression and clinicopathological factors in lung adenocarcinoma (*n* = 161).

Clinicopathological Characteristics		MTUS-1 Expression		
		Low Group (*n* = 74) (%)	High Group (*n* = 87) (%)	*p*-Value
Age	Mean (±SD *)	63.2 ± 9.9	61.3 ± 9.6	0.165 ^†^
Sex	Male	38 (51.4)	36 (41.4)	0.268
	Female	36 (48.6)	51 (58.6)	
Tumor size (cm)	Mean (±SD)	3.4 ± 2.2	2.5 ± 1.7	<0.001 ^†^
Histologic grade	Grade 1	7 (9.5)	24 (27.6)	0.007
	Grade 2, 3	67 (90.5)	63 (72.4)	
Pleural invasion	Present	25 (33.8)	25 (28.7)	0.604
	Absent	49 (66.2)	62 (71.3)	
Lymphovascular invasion	Present	38 (51.4)	27 (31.0)	0.014
	Absent	36 (48.6)	60 (69.0)	
Perineural invasion	Present	16 (21.6)	12 (13.8)	0.272
	Absent	58 (78.4)	75 (86.2)	
T stage	Tis, T1	29 (39.2)	49 (56.3)	0.044
	T2, T3, T4	45 (60.8)	38 (43.7)	
N stage	N0	44 (59.5)	69 (79.3)	0.010
	N1, N2, N3	30 (40.5)	18 (20.7)	
8th AJCC * Stage	0, I	36 (48.6)	62 (71.3)	0.006
	II, III	38 (51.4)	25 (28.7)	
Ki-67 proliferation index	Mean (±SD)	19.2 ±15.6	12.7 ± 14.1	<0.001 ^†^

* AJCC: American Joint Committee on Cancer; SD, standard deviation; † Mann–Whitney U test.

**Table 3:** Cox regression analysis of variables for predicting prognosis of lung adenocarcinoma.

Variables	Disease-Free Survival			Cancer-Specific Survival		
	HR *	(95% CI *)	*p*-Value	HR	(95% CI)	*p*-Value
**Univariable Analysis**						
Age	0.98	(0.95–1)	0.320	1	(0.99–1.1)	0.260
Sex	0.62	(0.34–1.1)	0.110	0.57	(0.31–1)	0.066
Tumor size	1.3	(1.2–1.4)	<0.001	1.2	(1.1–1.4)	<0.001
Histologic grade (1, 2, 3)	2.5	(1.5–4.2)	<0.001	2	(1.2–3.4)	0.012
Pleural invasion (absent vs. present)	1.5	(0.76–2.8)	0.260	2	(1–3.9)	0.038
Lymphovascular invasion (absent vs. present)	3.2	(1.7–6)	<0.001	3	(1.6–5.8)	<0.001
Perineural invasion (absent vs. present)	2.2	(1.1–4.2)	0.019	2.7	(1.4–5.3)	0.003
T stage (Tis, T1 vs. T2, T3, T4)	2.3	(1.2–4.2)	0.009	2.3	(1.2–4.3)	0.011
Lymph node metastasis (absent vs. present)	3.5	(2–6.4)	<0.001	4	(2.1–7.4)	<0.001
8th AJCC * stage (0, I vs. II, III)	3.7	(2–6.9)	<0.001	5.9	(2.8–12)	<0.001
Ki-67 proliferation index	1	(1–1)	0.019	1	(1–1)	0.030
MTUS1 expression (high vs. low)	2.6	(1.4–4.8)	0.003	2.4	(1.3–4.6)	0.007
**Multivariable analysis**						
Tumor size	1.3	(1.1–1.5)	0.001	1.2	(1.1–1.4)	0.005
Histologic grade (1, 2, 3)	1.0	(0.3–3.2)	0.974	2.1	(0.5–9.4)	0.333
Lymphovascular invasion (absent vs. present)	2.4	(1.1–4.9)	0.021	2.2	(1.0–4.6)	0.045
Perineural invasion (absent vs. present)	1.6	(0.7–3.5)	0.235	1.5	(0.7–3.3)	0.285
T stage (Tis, T1 vs. T2, T3, T4)	0.7	(0.3–1.6)	0.406	0.5	(0.2–1.3)	0.170
Lymph node metastasis (absent vs. present)	1.4	(0.5–3.9)	0.536	1.0	(0.4–2.6)	0.919
8th AJCC stage (0, I vs. II, III)	1.1	(0.3–3.4)	0.888	3.1	(1.0–9.7)	0.048
Ki-67 proliferation index	1.0	(1.0–1.0)	0.180	1.0	(1.0–1.0)	0.311
MTUS1 expression (high vs. low)	1.7	(0.9–3.3)	0.135	1.4	(0.7–2.8)	0.341

* AJCC: American Joint Committee on Cancer; HR, hazard ratio; CI, confidence interval.

## Supplementary Materials

The following are available online at https://www.mdpi.com/article/10.3390/diagnostics11071250/s1, Figure S1: “Boxplot of MTUS1 expression by immunohistochemistry (H-score) in different AJCC stage groups”, Figure S2: “Boxplot of MTUS1 expression by immunohistochemistry (H-score) in the three histologic grade groups”, Figure S3: “Comparison of survival curves according to MTUS1 expression by immunohistochemistry in early and late stage lung adenocarcinoma patients”, Table S4: “Comparison of survival curves according to MTUS1 expression by immunohistochemistry in lung adenocarcinoma patients who received surgical treatment only, and in patients who received adjuvant therapies”.
