# Supplementary material for: Low-Level Expression of MTUS1 Is Associated with Poor Survival in Patients with Lung Adenocarcinoma
Source: Diagnostics (Basel). 2021 Jul 13;11(7):1250. doi: 10.3390/diagnostics11071250 (PMC8306423; doi:10.3390/diagnostics11071250)
Supplement: Supplementary file 1 [file diagnostics-11-01250-s001.zip › diagnostics-1261625-supplementary.pdf]

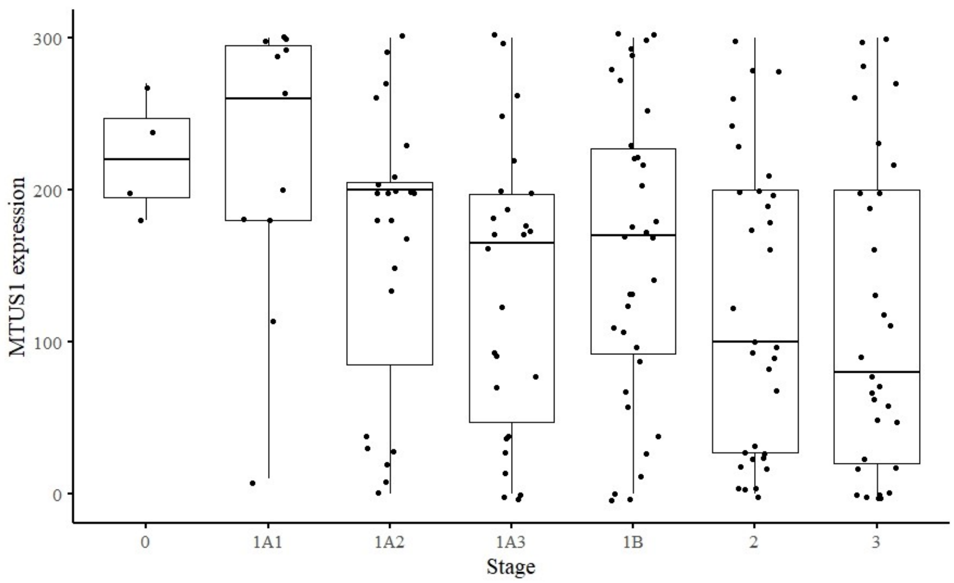

Supplementary Figure S1. Boxplot of MTUS1 expression in different AJCC stage groups. The median value of MTUS1 expression was highest in the 1A1 group (*n* = 11).

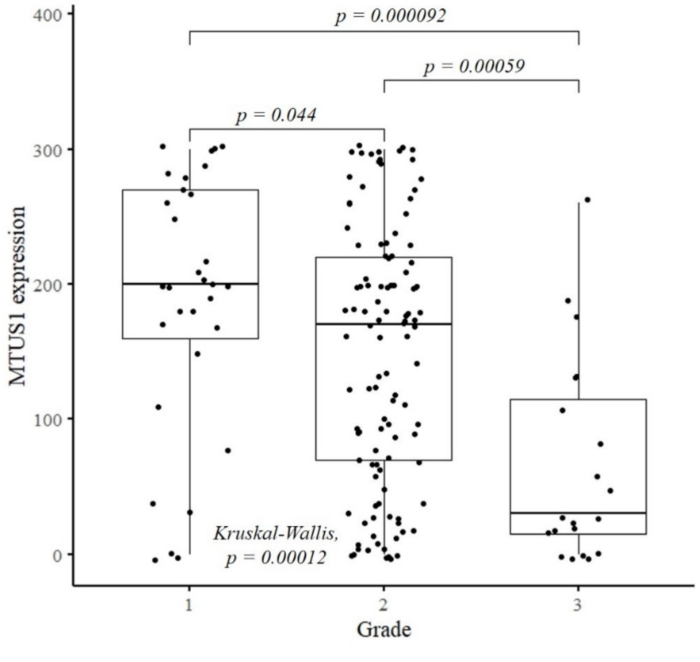

Supplementary Figure S2. Boxplot of MTUS1 expression in the three histologic grade groups. The means were significantly different among the three groups (Kruskal-Wallis test), and also between each pair of groups (Wilcoxon test adjusted by BH method).

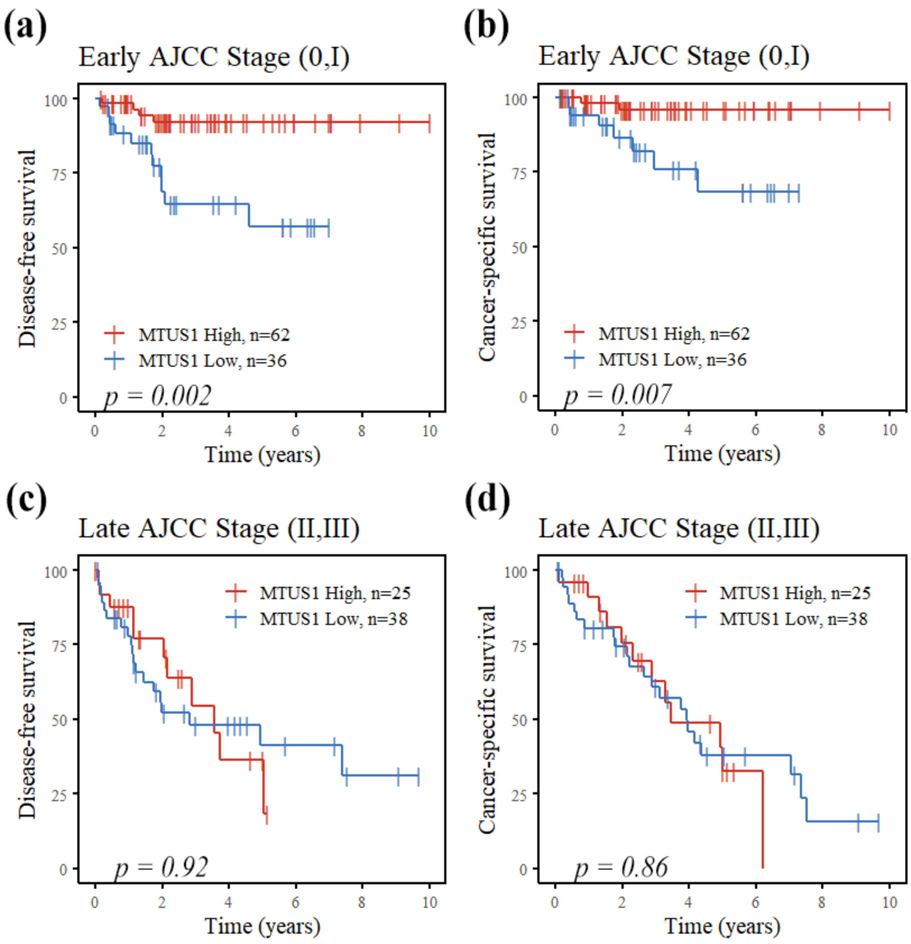

Supplementary Figure S3. Comparison of survival curves according to MTUS1 expression by immunohistochemistry in early and late stage lung adenocarcinoma patients. In early lung adenocarcinoma patients (AJCC stage 0 and I, $n = 98$), DFS (a) and CSS (b) according to MTUS1 expression showed significant differences ($p = 0.002$ and $p = 0.007$, respectively). However, patients with more advanced stages (AJCC stage II and III, $n = 63$), DFS (c) and CSS (d) according to MTUS1 expression showed no significant difference ($p = 0.92$ and $p = 0.86$, respectively) (Kaplan-Meier method with log-rank test).

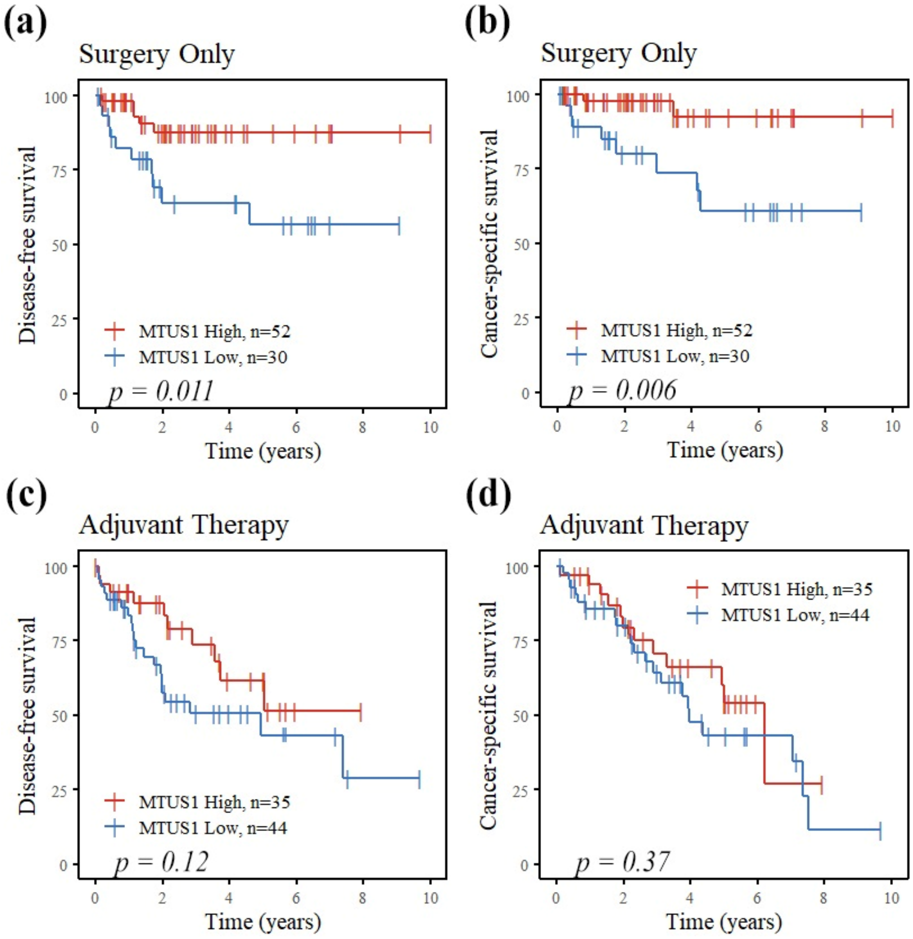

Supplementary Figure S4. Comparison of survival curves according to MTUS1 expression by immunohistochemistry in lung adenocarcinoma patients who received surgical treatment only, and in patients who received adjuvant therapies. For patients who have only had surgical treatment ($n = 82$), DFS (a) and CSS (b) according to MTUS1 expression showed significant differences ($p = 0.011$ and $p = 0.006$, respectively). However, for patients have received additional treatment ($n = 79$), DFS (c) and CSS (d) according to MTUS1 expression showed no significant difference ($p = 0.12$ and $p = 0.37$, respectively) (Kaplan-Meier method with log-rank test).
